# Thiamine supplementation in septic shock patients: still looking for the target population

**DOI:** 10.1186/s13054-024-05012-z

**Published:** 2024-07-08

**Authors:** Amanda Gomes Pereira, Nara A. Costa, Mariana B. de Moraes, Marina P. Okoshi, Leonardo A. M. Zornoff, Paula S. Azevedo, Marcos F. Minicucci, Sérgio A. R. de Paiva, Bertha F. Polegato

**Affiliations:** 1 Department of Internal Medicine, Botucatu Medical School, São Paulo State University (UNESP), Av. Prof. Mario Rubens Guimaraes Montenegro, S/N, UNESP - Campus de Botucatu, Botucatu, SP 18618 687 Brazil; 2 Faculty of Nutrition, Federal University of Goiás (UFG), Goiânia, Brazil

## Dear editor,

In recent years, there has been a growing interest in thiamine as a potential adjunctive therapy in septic shock, given its key role in the maintenance of cellular metabolism and energy production, being involved in various biological processes [1]. It is supposed that treatment of thiamine deficiency could mitigate alterations associated with organ dysfunction and lead to better outcomes in this critical population.

In light of this, Donnino and colleagues have been at the forefront of the efforts to provide clinical data regarding the efficacy of thiamine administration across different outcomes after sepsis [2–7]. We read with great interest the recent study published by his group, in which the authors conducted a post hoc analysis focusing on the effects of thiamine versus placebo on kidney protection. Vine et al. [8] included a cohort of 158 patients with septic shock from their two previous phase II clinical trials. The primary outcome of the study was the patients’ condition at the time of hospital discharge, which was defined as being alive and RRT (Renal Replacement Therapy)-free at the time of hospital discharge. They reported that thiamine administration was associated with higher odds of being alive and RRT-free (adjusted odds ratio [aOR] 2.05 [95% confidence interval (CI) 1.08–3.90]) and not needing RRT (aOR 2.59 [95% CI 1.01–6.62]). Importantly, the effect was more pronounced in the thiamine-deficient patients (considered as < 8 nmol/L).

Similarly, we performed a sub-analysis of our previous randomized pilot trial [9] to explore the effects of thiamine on renal outcomes. The cohort included a total of 108 patients with septic shock at Intensive Care Unit (ICU) admission who were not receiving RRT at the time of enrolment (51 in the thiamine group and 57 in the placebo group). In contrast to the findings of Vine et al. [8], our analysis revealed no difference between the thiamine and placebo groups in the need for RRT, even after adjusting for age, sex, creatinine, and APACHE II (Table 1). Additionally, we performed other analyses excluding patients with acute kidney injury (AKI) at ICU admission. In this analysis, we had only 27 patients in the placebo group and 19 in thiamine group. Despite that, thiamine administration was not able to reduce AKI or RRT (Tables 2 and 3, respectively).

**Table 1:** Logistic regression model for the prediction of RRT in 108 patients with septic shock

Variable	OR	CI 95%	*p*
RRT*	1.923	0.884–4.184	0.099
RRT**	1.916	0.667–5.506	0.227
RRT***	1.861	0.688–5.037	0.221

*Unadjusted **Adjusted by age, sex, creatinine and APACHE II ***Adjusted by creatinine

**Table 2:** Logistic regression model for the prediction of AKI in 43 patients with septic shock

Variable	OR	CI 95%	*p*
AKI*	2.222	0.600–8.237	0.232
AKI**	2.128	0.544–8.319	0.278

*Unadjusted **Adjusted by age, sex and APACHE II

**Table 3:** Logistic regression model for the prediction of RRT in 43 patients with septic shock

Variable	OR	CI 95%	*p*
RRT*	8.667	0.873–86.062	0.065
RRT**	5.495	0.482–62.682	0.170

*Unadjusted **Adjusted by age, sex and APACHE II

However, some points are important to be highlighted. In our analysis, the thiamine group showed a higher incidence of AKI at baseline compared to the placebo group (87% vs 64%, *p* = 0.068), although this difference was not statistically significant. Interestingly, creatinine was positively correlated with thiamine levels on baseline (*r* = 0.646, *p* < 0.001). The reduced glomerular filtration capacity, as indicated by the elevated creatinine concentration, may have led to a decreased urinary excretion of thiamine, resulting in a thiamine deficiency rate of only 8.6% (n = 9) in our sample. This is notably lower than the 29% reported by Vine et al. [8], who suggested that thiamine deficiency-patients would benefit most from thiamine administration. It is important to note that our relatively small sample size could increase the risk of a type II error and may have influenced the results.

The promising potential of using thiamine to manage septic shock is primarily grounded in the physiological plausibility of treating the deficiency of this essential vitamin. However, the clinical evidence is currently insufficient and inconclusive both in the general and target septic shock population. Further research is still needed to refine methods for accurately measuring thiamine levels and to identify which patient subpopulations might benefit most from thiamine administration.

Therefore, we can conclude that, to date, taken together, study results have not consistently demonstrated benefits with thiamine supplementation in patients with septic shock, including decreased mortality and renal protection. However, in patients with thiamine deficiency, supplementation could induce beneficial effects.

## Data Availability

The datasets utilized and/or analyzed during this study can be obtained from the corresponding author upon reasonable request.
